# Pharmacogenomics: Challenges and Future

**DOI:** 10.3390/genes15060714

**Published:** 2024-05-30

**Authors:** Mariamena Arbitrio

**Affiliations:** Institute of Biomedical Research and Innovation (IRIB-CNR), Section of Catanzaro, 88100 Catanzaro, Italy; mariamena.arbitrio@irib.cnr.it

Over the last few decades, the implementation of pharmacogenomics (PGx) in clinical practice has improved tailored drug prescriptions. PGx investigates how individual genetic makeup influences variability in drug response in terms of pharmacokinetics (PK) and pharmacodynamics (PD), adverse drug reactions, and clinical outcomes. Although the FDA provides recommendations for drug absorption, distribution, metabolism, and elimination (ADME)-related genes for over 200 drugs in several therapeutic areas, especially for cancer, there are several barriers to the full implementation of PGx. This Special Issue contains six research articles and five reviews, one of which is a systematic review. The manuscripts cover several topics of interest in the PGx field, including bioinformatic approaches for data analysis and biomarker discovery and validation.

Luzum et al. [1] presented work on interindividual variability in beta blocker treatment in black and white patients with heart failure and reduced ejection fraction (HFrEF). The authors discussed using an unbiased, discovery-based, genome-wide association study (GWAS) to identify loci influencing beta blocker survival benefits in these patients; they identified many genetic variants correlated with such benefits, but none were validated in an independent dataset. However, one variant (rs16844448) in *LRP1B* was found to be suggestive of an association with beta blocker survival benefit in black patients with HFrEF. The conclusion of this work suggests that larger cohorts or alternative approaches, such as polygenic scores, are needed to discover and validate novel predictive/prognostic genetic variant(s).

Polymorphic variants and epigenetic factors, such as DNA methylation sites, significantly influence interindividual variability in drug response and disease risk, providing insights into potential therapeutic targets for several disorders, particularly cancer. In a retrospective study, Martínez-Iglesias et al. [2] examined the global DNA methylation levels (5 mC) of patients affected by hypovitaminosis, as well as neuropsychiatric, cerebrovascular, and neurodegenerative disorders, to evaluate 5 mC’s role as a diagnostic biomarker for several diseases; the evaluation was performed in serum samples from patients during initial and follow-up visits and identified two groups of patients, one in which 5 mC levels improved during the clinical follow-up (Group A) and another (Group B) with lower 5 mC levels during the follow-up visit. This study aimed to investigate if the discrepancy in the treatment response highlighted in these two groups of patients was correlated with 5 mC levels, compared to other biochemical and psychometrical criteria, to determine which patients responded better to treatment and exhibited improved outcomes. The authors concluded that patients with hypovitaminosis and/or with better neuropsychometric scores had higher 5 mC values following treatment. Therefore, 5 mC could be proposed as a diagnostic biomarker across different pathologies.

Polymorphic variants in ADME genes involved in the activation of prodrugs can significantly modify the PK of several anticancer drugs, as in the case of 5-fluorouracil, mercaptopurine, and irinotecan (DPYD, TPMT, and UGT1A1). In 76 healthy Japanese subjects, Ito et al. [3] reported data regarding the role of Cathepsin A (CatA) gene polymorphisms in influencing the expression level of CatA protein and drug-metabolizing activity. Nine genetic polymorphisms were identified in this gene, which plays an important role in the metabolism of tenofovir alafenamide (TAF/GS-7340), used in antiretroviral therapy for human immunodeficiency virus (HIV) and hepatitis B, and sofosbuvir (SOF/GS-7977/PSI-7977), used for hepatitis C virus.

It was found that variants 1 and 2 are the major transcripts of CatA in human lymphocytes. The conclusions of this work suggest that genetic polymorphisms can influence the drug metabolic activity of CatA.

In the era of precision medicine, identifying patients who can benefit from specific therapies prescribed on the basis of known pharmacogenomic interactions, especially in cancer, is of crucial importance. Fasola et al. [4] provided a methodological framework to discover pharmacogenomic interactions based on Random Forests, an ML methodology helpful for mining and graphically exploring such interactions. Open R code was made available for implementation on ordinary computer platforms. This methodology is carried out with ordinary computational resources and uses R version 4.0.2 (R Foundation for Statistical Computing, Vienna, Austria) as the reference software. Two databases, from the Cancer Cell Line Encyclopedia (CCLE) and the Genomics of Drug Sensitivity in Cancer (GDSC) projects, were matched. The authors identified well-known drug–gene associations and provided clues to discover novel pharmacogenomic interactions.

All genomic data identified in PGX studies need to be analyzed through a stringent methodology based on bioinformatic approaches. Milano et al. [5] reported an exploratory application of network and pathway analysis for PGx data. Multilayer networks represent a more comprehensive, realistic, and powerful strategy to model and analyze complex datasets with multiple types of interactions compared to traditional ones, while network analysis applied to PGx allows gaining insights into interactions between genes, drugs, and diseases. The integration of these types of approaches allows us to uncover complex relationships and identify several key genes to use in pathway enrichment analysis, in order to discover biological pathways involved in drug response and adverse reactions. The authors identified several genes that formed the top community, applying pathway enrichment analysis (PEA) to determine the biological function affected by the selected genes. The selected genes interfere in the activity of the protein metabolism pathway associated with adverse or normal drug responses, as well as the progression or decline of disease. Also, an understanding of the considerable amount of data derived from PGx research tools, such as DNA microarrays, can be gained through several biostatistical, ML, and statistical methods to allow a better interpretation of the results’ biological meaning. Agapito et al. [6] described a step-by-step protocol for the Python clustering analysis of omics data in order to identify biomarkers and yield computational predictive models through the K-means and DBSCAN algorithms. Moreover, through an unsupervised learning approach, the authors provided some best practices and tips to overcome issues in omics dataset analysis.

Another contribution, by Ciluffo et al. [7], provided an overview of the importance of ML tools in the analysis and interpretation of PGx data over the past 10 years. In this narrative review, the authors discussed supervised machine learning (SML) and unsupervised machine learning (UML). The first is applied when prediction is the focus of the research, while UML tools are used when the outcome is not known and the goal of the research is unveiling the underlying structure of the data. In this Special Issue, alongside the above-mentioned papers are also three reviews on the PGx of dementia, childhood asthma, and the flavoenzyme N-ribosyldihydronicotinamide (NRH): quinone oxidoreductase 2 (NQO2). Vuic et al. [8] presented research on neurodegenerative diseases characterized by dementia, usually affecting the elderly, in which significant interindividual variability in drug response and the development of adverse drug effects influence quality of life and patient outcomes. Advances in pGx could allow the personalization and optimization of dementia pharmacotherapy by increasing efficacy and safety via the prediction of clinical outcomes. High interindividual variability in drug response is also common in asthmatic patients, including in childhood, and genetic makeup could partly explain this variability. In addition, genetic variants, inherited from a specific ancestry associated with disease severity or response to treatment, could influence the differences in response to asthma medications of individuals from different populations and ethnic groups. Ferrante et al. [9] discussed this and the application of PGx in childhood asthma practice, highlighting the challenges and evidence gaps. Janda et al. [10] reported a detailed overview of the literature on NQO2, an enzyme that catalyzes two-electron reductions of quinones involved in the metabolism of biogenic and xenobiotic quinones, including a wide range of antitumor drugs, with both toxifying and detoxifying functions. NQO2 may also play important roles in the regulation of oxidative stress, inflammation, and autophagy, with implications in carcinogenesis and neurodegeneration. Polymorphic variants in this enzyme can alter cancer susceptibility and progression, modify the response to chemotherapy, and may predispose to Parkinson’s disease and other neurological dysfunctions.

In the final work, Lumkul et al. [11] perform a systematic review and meta-analysis of genetic effects regarding beta-lactam (BL) antibiotics-induced hypersensitivity to aid the determination of disease-related genotypes, which could potentially facilitate allergy profiling in patients.

All articles included in this Special Issue demonstrate how PGx or genomic data analysis via bioinformatic approaches could play a key role in personalizing therapy. In future, the opportunity to further unveil the relationship between ADME genes and drug response, as well as identifying new genes involved in the effects and/or toxicity of anticancer drugs or the repurposing of drugs, could open up new avenues for fully implementing PGx in the clinical setting. We are grateful to all the authors included in this issue and to all the reviewers for their excellent feedback, without which we would not have been able to encourage researchers in the field to continue the great work of identifying polymorphic ADME gene variants’ clinical implications in therapy and disease susceptibility.
